# Mobile educational tool based on augmented reality technology for tooth carving: results of a prospective cohort study

**DOI:** 10.1186/s12909-023-04443-6

**Published:** 2023-06-21

**Authors:** Eun-Jeong Lim, Yi-Seul Kim, Ji-Eun Im, Jae-Gi Lee

**Affiliations:** 1 Department of Dental Hygiene, Graduate School of Namseoul University, Cheonan, Republic of Korea; 2 Department of Dental Hygiene, Seoyeong University, Gyeonggi, Republic of Korea; 3 Department of Dental Hygiene, College of Health and Health care, Namseoul University, Cheonan, Republic of Korea

**Keywords:** Augmented reality, Dental carving, Dental education, Mobile device, Tooth morphology

## Abstract

**Background:**

Augmented reality (AR) technology has been shown to be effective in displaying information and presenting three-dimensional objects. Although AR applications are commonly used by learners via mobile devices, plastic models or two-dimensional images are still commonly used in tooth carving practice. Learners practicing tooth carving face a challenge due to the three-dimensional features of teeth as there is a lack of tools available that provide sequential guidance. In this study, we developed an AR-based tooth carving practice tool (AR-TCPT) and compared it to a plastic model to evaluate its potential as a practice tool as well as its user experience.

**Methods:**

To model tooth carving, we created a three-dimensional object from sequential steps that included the maxillary canines and maxillary first premolars (16 steps), mandibular first premolars (13 steps), and mandibular first molars (14 steps). Image markers, created using Photoshop software, were assigned to each tooth. An AR-based mobile application was developed using the Unity engine. For tooth carving, 52 participants were randomly assigned to a control group (n = 26; using a plastic tooth model) or an experimental group (n = 26; using the AR-TCPT). User experience was evaluated using a 22-item questionnaire. Data were comparatively analyzed using the nonparametric Mann–Whitney U test via the SPSS program.

**Results:**

The AR-TCPT detects image markers with the mobile device camera and displays three-dimensional objects for tooth fragmentation. Users can manipulate the device to view each step or examine the shape of a tooth. The results of the user experience survey revealed that the AR-TCPT experimental group scored significantly higher in tooth carving experience compared with the control group that used the plastic model.

**Conclusion:**

Compared with the conventional plastic model, the AR-TCPT provided a better user experience for tooth carving. The tool is highly accessible as it is designed to be used on mobile devices by users. Further studies are required to determine the educational impact of the AR-TCTP on quantitative scoring of carved teeth as well as individual user’s carving abilities.

## Background

Dental morphology and practice class is an essential part of the dentistry curriculum. This course provides theoretical and practical instruction on the morphology, function, and direct carving of tooth structure [1, 2]. The conventional teaching method involves theoretical learning followed by tooth carving based on the principles learned. Students use two-dimensional (2D) tooth images and plastic models to carve teeth on wax or plaster blocks [3–5]. Understanding tooth morphology is essential in prosthetic treatment and the creation of dental restorations in clinical practice. The proper relationship between the antagonist and proximal teeth, as indicated by their shape, is critical for maintaining occlusion and alignment stability [6, 7]. Although the dentristry curriculum helps students gain a comprehensive understanding of tooth morphology, they still face difficulties during the tooth carving process associated with conventional practice.

Beginner students in dental morphology practice face challenges interpreting and reproducing 2D images in three-dimensional (3D) form [8–10]. Tooth shapes are typically represented in 2D drawings or photographs, leading to difficulties in visualizing tooth morphology. Moreover, the pressure to complete tooth carving quickly within limited time and space, coupled with the use of 2D images, makes it difficult for learners to conceptualize and visualize 3D shapes [11]. Although plastic tooth models, available as partial completion or final form displays, aid learning, they have limited use, as commercialized plastic models are typically predefined and restrict practice options for instructors and learners alike [4]. Furthermore, these practice models are held by educational institutions and cannot be personally owned by students, leading to increased pressure to complete the pieces within the allotted class time. Instructors often guide a large number of students during the practice process, usually relying on conventional practice methods, which can lead to prolonged waiting times for instructor feedback on intermediate carving steps [12]. Therefore, a supplementary carving guide medium is needed to facilitate tooth carving practice and mitigate the limitations posed by plastic models.

Augmented reality (AR) technology has emerged as promising tool for enahncing learning experiences. By superimposing digital information onto the real environment, AR technology can provide learners with a more interactive and immersive experience [13]. According to Garzón [14], who drew on 25 years of experience in educational classification of AR spanning the first three generations, the use of cost-effective mobile devices and applications in 2nd generation AR (via mobile devices and applications) has significantly improved educational characteristics. Upon creation and installation, the mobile application enables the camera to recognize and display additional information about recognized objects, thereby enhancing user convenience [15, 16]. AR technology operates via quick response code or image marker recognition through a mobile device camera, displaying superimposed 3D information upon detection [17]. By manipulating the mobile device or image marker, users can easily and intuiviely observe and comprehend the 3D structure [18]. In the review of Akçayır and Akçayır [19], it was found that AR enhances “enjoyment” and succeeds in “raising the level of engagement” in pedagogy. Nevertheless, the technology can be “difficult for students to use” and result in “cognitive overload” due to data complexity, making supplementary learning guidance necessary [19–21]. Therefore, efforts must be made to enhance the educational value of AR by improving usability and reducing task complexity overload. These factors must be considered when creating educational media for tooth carving practice using AR technology.

To effectively guide learners in tooth carving using AR media, a sequential process should be followed. This approach can help reduce variation and facilitate skill mastery [22]. Novice carvers can improve the quality of their work by following a digital step-by-step tooth carving process [23]. Indeed, a step-by-step learning method has been shown to be effective in acquiring carving skills within a short period of time and minimizing errors in the final wax form for restorations [24]. In the field of dental prosthetics, subdividing the engraving process applied to the tooth surface is an effective way to help learners enhance their skills [25]. The present study aims to develop a mobile-friendly AR-based tooth carving practice tool (AR-TCPT) and evaluate its user experience. Furthermore, the study compares the user experience of AR-TCPT with that of a conventional plastic model for tooth carving, with the goal of assessing the potential of AR-TCPT as a practice tool.

## Methods

### Research design

**Fig. 1:**
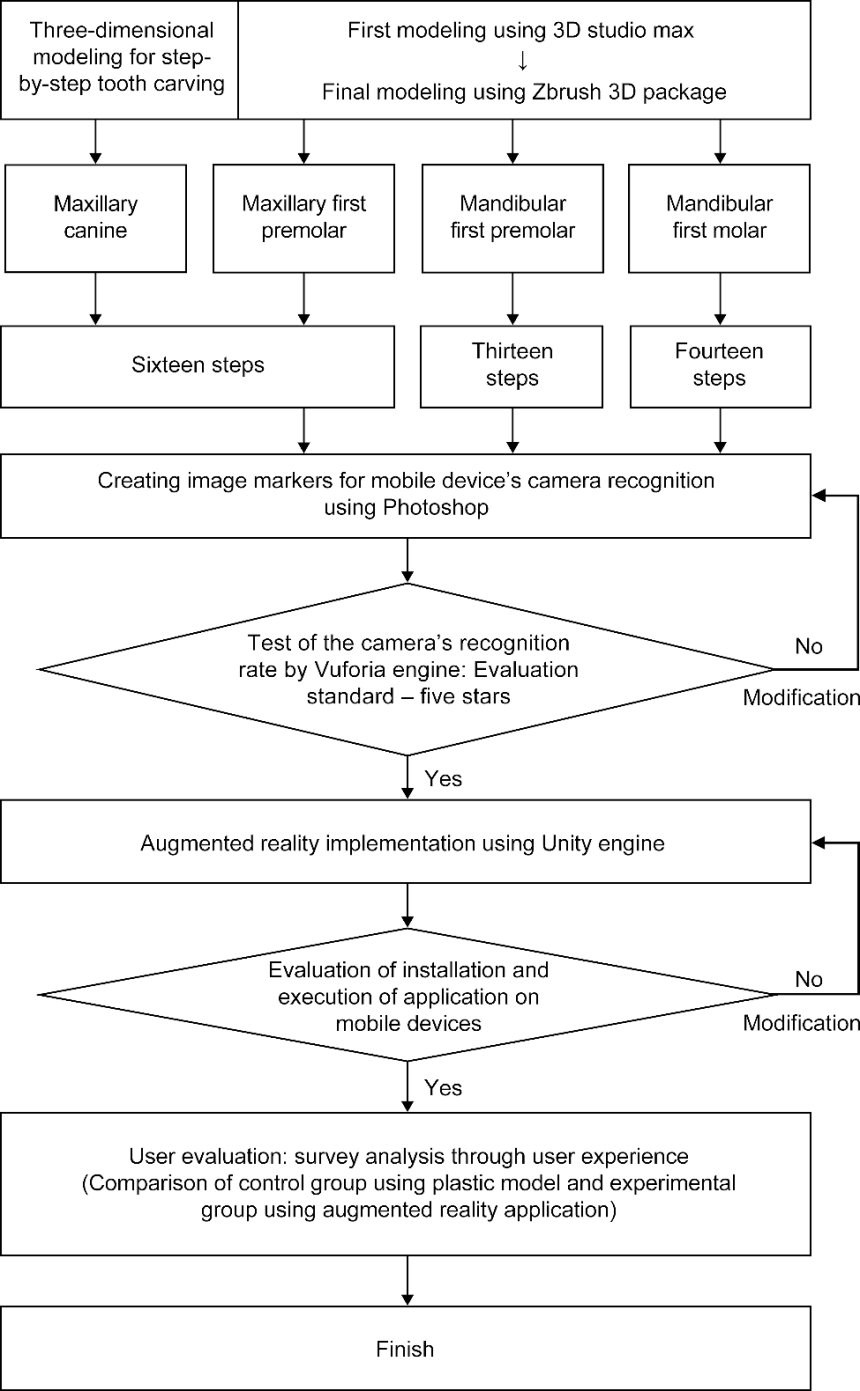
Development and user evaluation of an educational tool for tooth carving practice

The AR-TCPT was developed using AR technology for mobile devices. The tool was designed to create 3D models of the maxillary canine, maxillary first premolar, mandibular firt premolar, and mandibular first molar in a stepwise manner. Initial 3D modeling was completed using 3D Studio Max (2019, Autodesk Inc., USA), whereas final modeling was performed using the Zbrush 3D package (2019, Pixologic Inc., USA). Image markers were produced using Photoshop software (Adobe Master Collection CC 2019, Adobe Inc., USA) and were designed for stable recognition by mobile cameras, achieving a five-star rating in the Vuforia engine (PTC Inc., USA; http://developer.vuforia.com). The AR application was implemented using the Unity engine (2019.3.12., Unity Technologies, USA) and subsequently installed and operated on a mobile device. To evaluate the effectiveness of the AR-TCPT as a practice tool for tooth carving, a control group and experimental group were formed by randomly selecting participants from a class of dental morphology practice in 2023. Participants in the experimental group used the AR-TCPT, whereas those in the control group used the plastic model from a Tooth Carving Step Model Kit (Nissin Dental Inc., Japan). After completing the tooth carving task, user experiences for each practice medium were investigated and compared. The order of the research design is presented in Fig. 1. The study was conducted with the approval of the Institutional Review Committee of Namseoul University (IRB No: NSU-202210-003).

### 3D modeling following the tooth carving stages

**Fig. 2:**
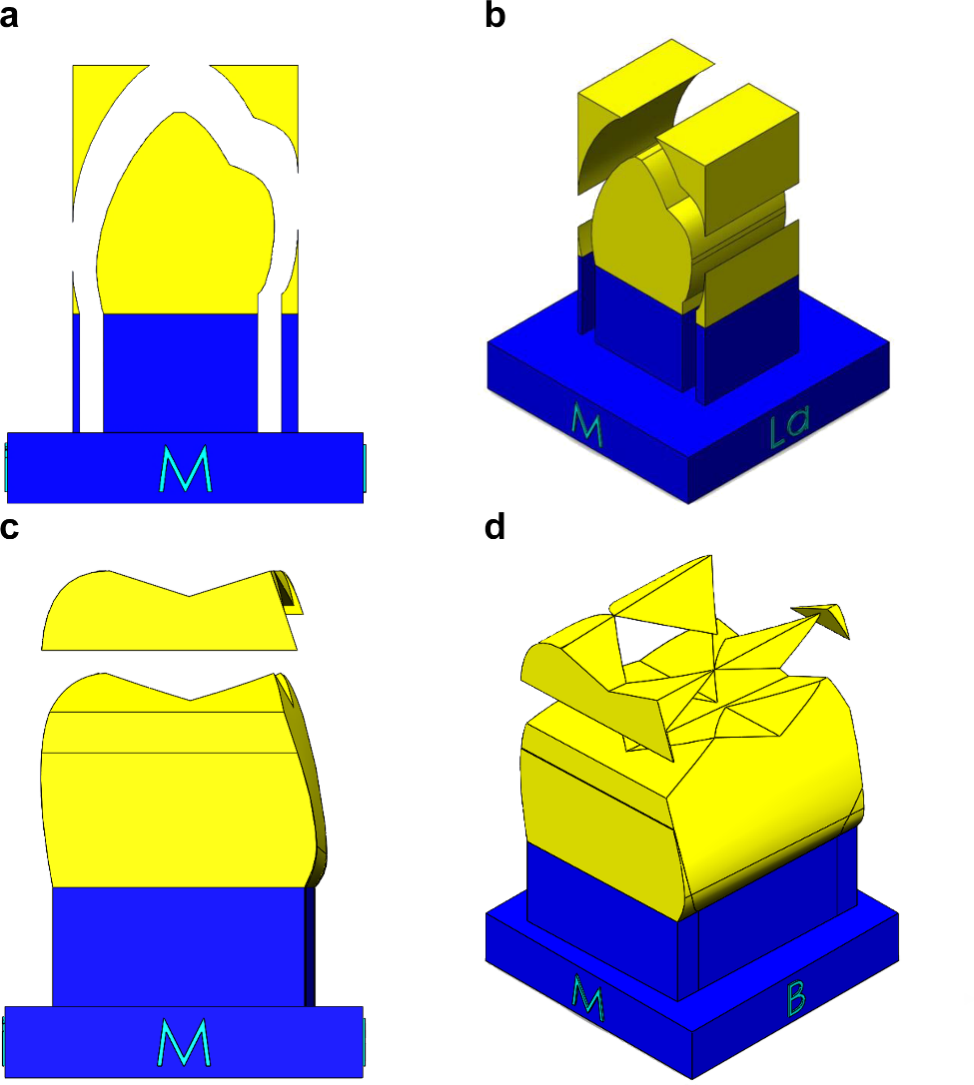
Primary modeling for creating three-dimensional objects in the step-by-step tooth carving process. **a**: mesial aspect of the maxillary first premolar; **b**: slightly superior and mesiolabial aspect of the maxillary first premolar; **c**: mesial aspect of the maxillary first molar; **d**: slightly superior and mesiobuccal aspect of the maxillary first molar. B, buccal; La, labial; M, mesial

**Fig. 3:**
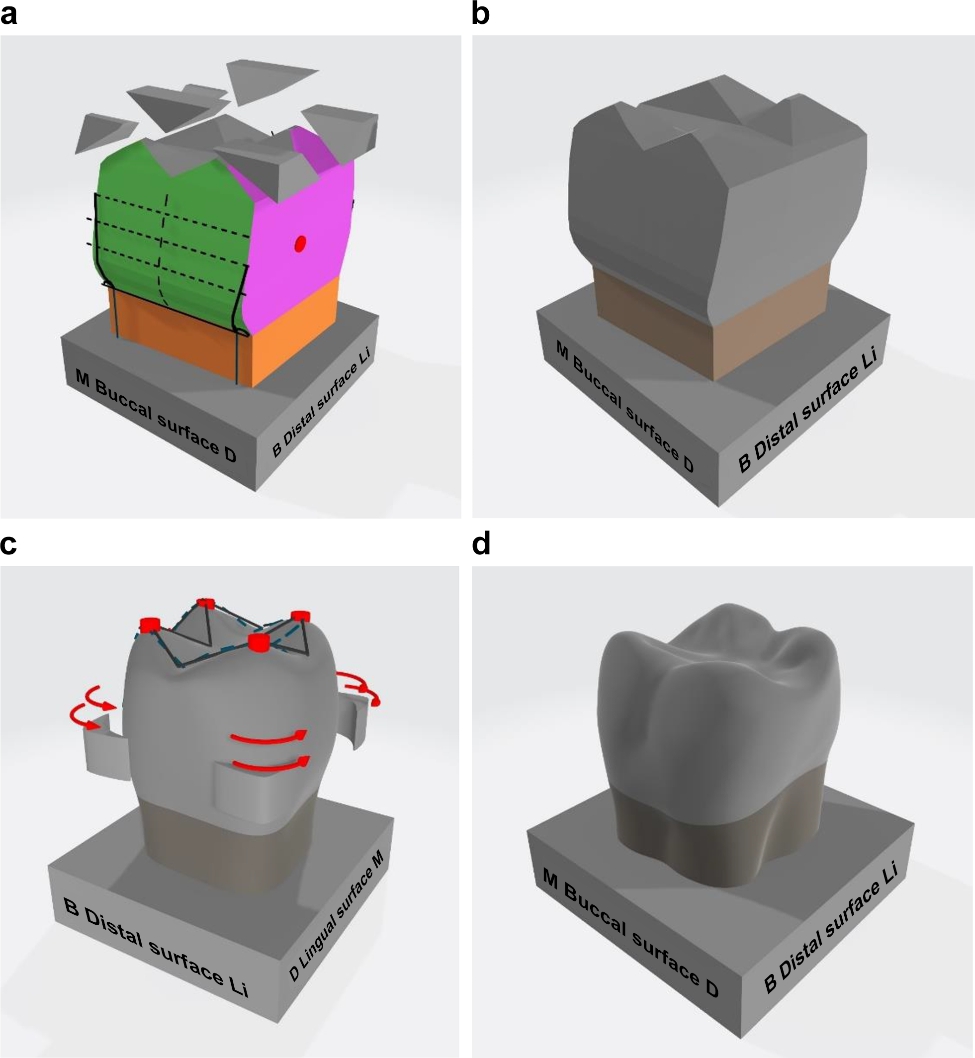
Three-dimensional (3D) objects representing the step-by-step tooth carving process. This photograph shows completed 3D objects following the maxillary first molar modeling process and provides detailed information and textures for each sequential step. The second 3D modeling data include the final 3D object augmented in the mobile device. The dotted line indicates equal divisions of the tooth, and a separate piece indicates that it must be removed before including a piece containing a solid line. The red 3D arrow indicates the direction of tooth carving, the red circle on the distal surface represents the contact area of the tooth, and the red cylinder on the occlusal surface indicates the cusp. **a**: Dotted line, solid line, red circle on the distal surface, and steps indicating the wax block to be separated. **b**: Approximate completion of the maxillary first molar. **c**: Detailed view of the maxillary first molar, with the red arrow indicating the direction of tooth carving and the separated piece, red cylindrical cusp, and shape of the solid line indicating the part to be carved on the occlusal surface. **d**: Completed maxillary first molar

3D modeling was used to display the morphological features of the teeth’s prominent and depressed structures on the mesial, distal, buccal, lingual, and occlusal surfaces in a sequential manner during the carving process. The teeth were modeled in 16 steps for the maxillary canine and maxillary first premolar, 13 steps for the mandibular first premolars, and 14 steps for the mandibular first molars. The primary modeling depicted the parts to be deleted and preserved, following the order of the tooth pieces, as shown in Fig. 2. The final modeling sequence of teeth is presented in Fig. 3. In the final modeling, the texture, ridges, and depressed structures of teeth were described, and pictorial information was included to guide the carving process and highlight structures requiring careful attention. At the beginning of the carving stage, each surface was color-coded to indicate its direction, and the wax block was marked with solid lines to indicate the parts to be removed. The mesial and distal surfaces of the teeth were marked with red dots to identify tooth contact points, which were to be preserved as prominences and not removed during the carving process. On the occlusal surfaces, red dots marked each cusp as preserved, and a red arrow indicated the carving direction during wax block carving. The 3D modeling of preserved and removed parts allowed for confirmation of the morphology of removed parts in the subsequent step of wax block carving.

### Image markers and implementation of AR

**Fig. 4:**
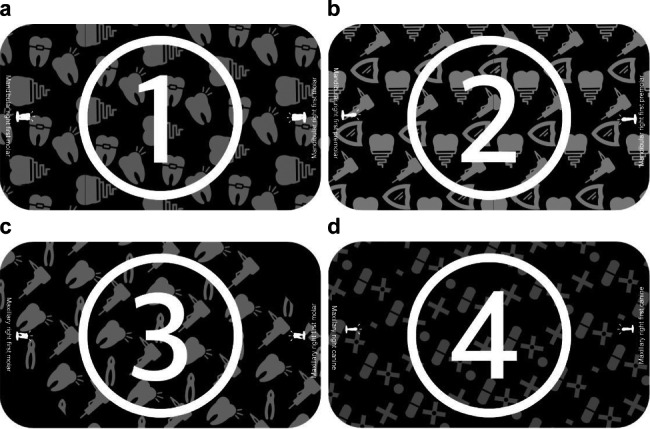
Image markers. These photographs show the image markers used in this study, which are recognized by the camera of the mobile device according to the type of tooth (number in each circle). **a**: Mandibular first molar; **b**: mandibular first premolar; **c**: maxillary first molar; **d**: maxillary canine

To facilitate the recognition of sequential carving steps using a mobile device, four image markers were prepared for the mandibular first molar, mandibular first premolar, maxillary first molar, and maxillary canine. The image markers were designed using Photoshop software (2020, Adobe Co., Ltd., San Jose, CA), and they distinguished each tooth using a circle–number symbol and a background of repeated patterns, as shown in Fig. 4. The Vuforia engine (AR marker production software) was used to produce high-quality image markers, and after evaluating the single image type recognition rate with five stars, the image markers were designed and saved using the Unity engine. The 3D tooth model was step-by-step linked to the image marker, and its position and size were determined based on the marker. Using the Unity engine, and Android application that could be installed on mobile devices.

### Recruitment of participants

**Fig. 5:**
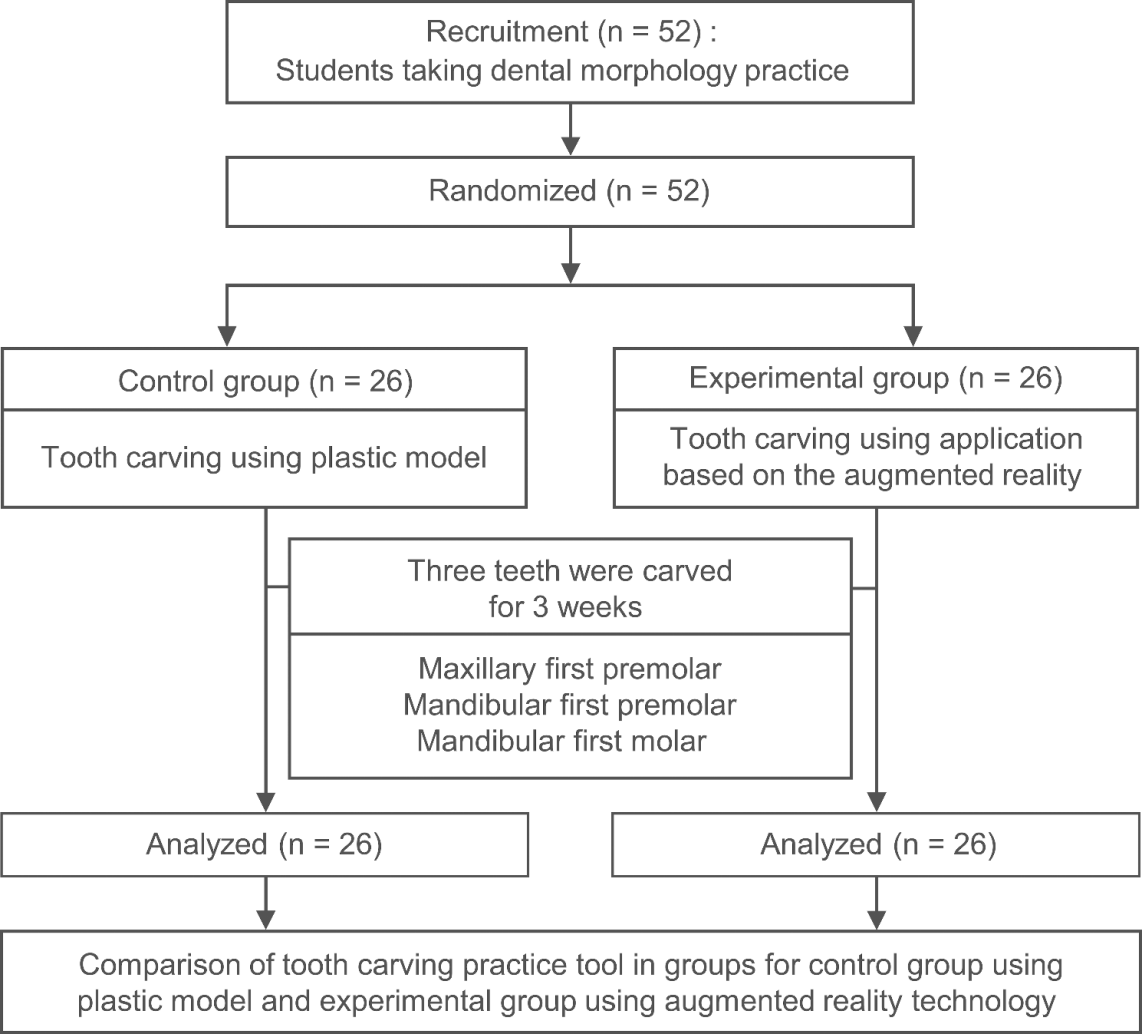
Research design for investigating the experiences of participants using plastic models and augmented reality applications

Participants were recruited from the first-year dental morphology practice class at the department of dental hygiene at Seoyeong University in Gyeonggi-do. Potential participants were informed of the following: (1) participation was voluntary, without financial or academic grade reward; (2) the control group would use a plastic model while the experimental group would use the AR mobile application; (3) the experiment would take place over three weeks and involve carving three teeth; (4) Android users would receive an application installation link, whereas iOS users would be lent Android devices with AR-TCPT installed; (5) AR-TCTP would function in the same manner in both systems; (6) the control and experimental groups would be randomly assigned; (7) tooth carving would take place in separate labs; (8) after completion of the experiment, a survey consisting of 22 items would be conducted; and (9) the control group could use the AR-TCPT after the experiment. In total, 52 participants volunteered, and online consent forms were obtained from each participant. The control group (n = 26) and experimental group (n = 26) were randomly assigned using the random function in Microsoft Excel (2016, Redmond, the USA). Figure 5 shows the participant recruitment and experimental design in a flowchart.

### Participant user experiences and comparative questionnaire

The experimental and control group carved three teeth each for three weeks, beginning on March 27, 2023, using the AR-TCPT and plastic model, respectively. Participants carved premolars and molars, including the mandibular first molar, mandibular first premolar, and maxillary first premolar, all with complex morphological features. The maxillary canines were not included in the carving. Participants were given a weekly time limit of 3 h to carve one tooth. After completing one tooth, the plastic model and image marker of the control and experimental groups, respectively, were retrieved. The 3D object of the tooth was not augmented via the AR-TCTP without image marker recognition. To prevent the use of other practice tools, the experimental and control groups practiced tooth carving in separate spaces. Feedback on tooth shape was provided for three weeks after the experiment concluded to limit the influence of instructor guidance. After completing the mandibular first molar carving in the third week of April, a questionnaire survey was conducted. The modified questionnaire of Saunders et al. [26] with 23 questions was used by Alfalah et al. [27] to assess the differences in heart shape between practice tools. However, in the present study, one item for direct manipulation per layer was excluded from the questionnaire items of Alfalah et al. [27]. The 22 items used in the current study are presented in Table 1. In the control and experimental groups, Cronbach’s α values were 0.587 and 0.912, respectively.

**Table 1:** Questionnaire items for comparing experiences of the control group (plastic model) and experimental group (AR-TCPT)

Questionnaire items	
Q1	It helps me better understand and memorize the step-by-step tooth carving process
Q2	It enhances the visualization of the step-by-step tooth carving process
Q3	It can clearly indicate the relative position among several structures
Q4	The step-by-step tooth carving process can be understood in an easy manner
Q5	It provides an opportunity to repeat a training task
Q6	It is available when required
Q7	I think it is a good learning tool
Q8	The tool is flexible for training
Q9	I can easily understand the step-by-step tooth carving process from different perspectives
Q10	The tooth structure can be understood in a short period
Q11	I can easily navigate through different parts of the tooth
Q12	It can reduce the time required to learn basic tooth morphology
Q13	It can help develop basic tooth carving skills before encountering actual patients
Q14	It can cover most of the tooth carving practice
Q15	I believe it can strengthen my intentions to learn
Q16	It reduces the use of textbooks as a learning method
Q17	The interaction is simple and clear while using the tool
Q18	I enjoy using the tool
Q19	It is easy to interact with others using this practice tool
Q20	I can clearly distinguish and observe the order of carving the teeth
Q21	This practice tool provides information to students regarding the components of tooth morphology for tooth carving
Q22	I can approach a tooth from different angles

AR-TCPT, augmented reality-based tooth carving practice tool; Q, question item

### Statistical analysis

SPSS statistical software (v25.0, IBM Co., Armonk, NY, USA) was used for data analysis. A significant two-tailed test was conducted at a significance level of 0.05. Fisher’s exact test was used to analyze general characteristics, such as gender, age, place of residence, and tooth carving experience, to confirm the distribution of these characteristics between the control and experimental groups. The survey data did not follow a normal distribution (p < 0.05), as evidenced by the results of a Shapiro–Wilk test. Therefore, the nonparametric Mann–Whitney U test was used to compare the control and experimental groups.

## Results

**Fig. 6:**
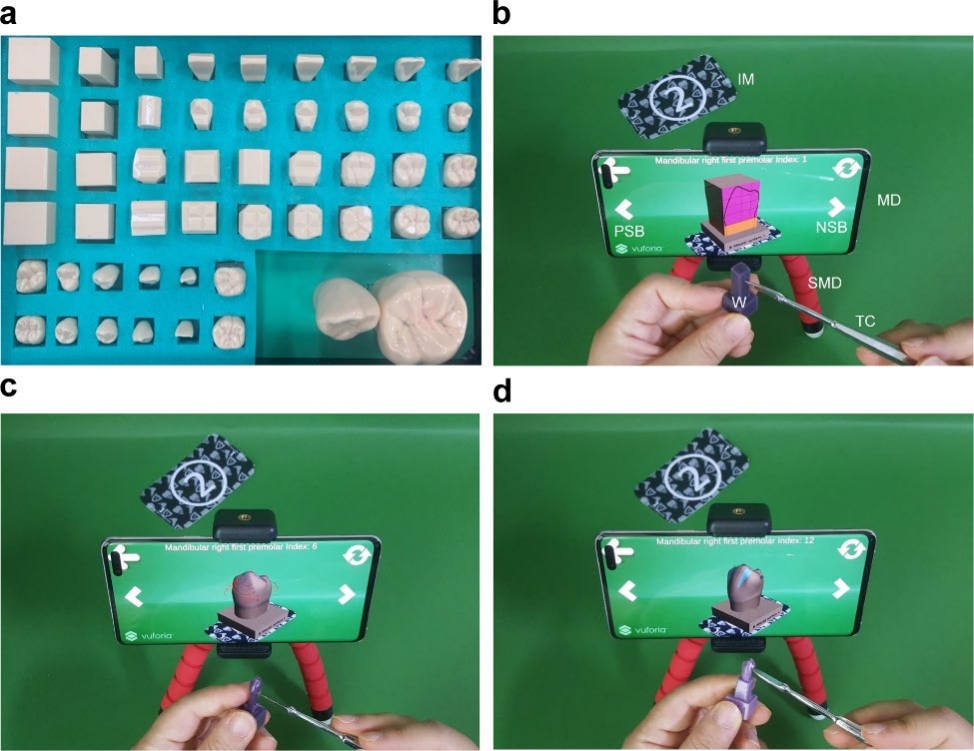
Tooth carving practice. This photograph shows a comparison between conventional tooth carving practice (TCP) using a plastic model and step-by-step TCP using an augmented reality-based tool. The learner can observe the carving step in three dimensions by pressing the next step and previous step buttons. **a**: Plastic model from a tooth carving step model kit. **b**: TCP using the augmented reality-based tool in the first stage of the mandibular first premolar. **c**: TCP using the augmented reality-based tool in the final stage of the mandibular first premolar. **d**: Process of identifying ridges and grooves. IM, image marker; MD, mobile device; NSB, next step button; PSB, previous step button; SMD, stand of mobile device; TC, tooth carver; W, wax block

The tools used by the participants for tooth carving practice are depicted in Fig. 6. Figure 6a shows a plastic model, and Fig. 6b–d shows the AR-TCPT used on a mobile device. The AR-TCPT used the device’s camera to recognize the image marker and display an augmented 3D tooth object on the screen, which participants could manipulate and observed in real-time. The mobile device’s next and previous step buttons allowed for a detailed observation of tooth carving steps and the morphological features of teeth. To carve the tooth, the AR-TCPT users compared the augmented 3D tooth model on the screen with a wax block in a sequential manner.

The randomly allocated participants in the two groups showed no significant differences in gender, age, place of residence, and tooth carving experience (p > 0.05). The control group comprised 96.2% females (n = 25) and 3.8% males (n = 1), whereas the experimental group solely consisted of females (n = 26). The control group had 61.5% (n = 16) 20-year-old participants, 26.9% (n = 7) 21-year-old participants, and 11.5% (n = 3) ≥ 22-year-old participants, whereas the experimental group comprised 73.1% (n = 19) 20-year-old participants, 19.2% (n = 5) 21-year-old participants, and 7.7% (n = 2) ≥ 22-year-old participants. In terms of the place of residence, 69.2% (n = 18) of the control group lived in Gyeonggi-do, whereas 23.1% (n = 6) lived in Seoul. In contrast, 50.0% (n = 13) of the experimental group lived in Gyeonggi-do, whereas 46.2% (n = 12) lived in Seoul. The percentages of the control group and the experimental group living in Incheon were 7.7% (n = 2) and 3.8% (n = 1), respectively. Out of the control group, 25 participants (96.2%) had no prior tooth carving experience. Similarly, 26 participants (100%) in the experimental group had no prior experience in tooth carving.

**Fig. 7:**
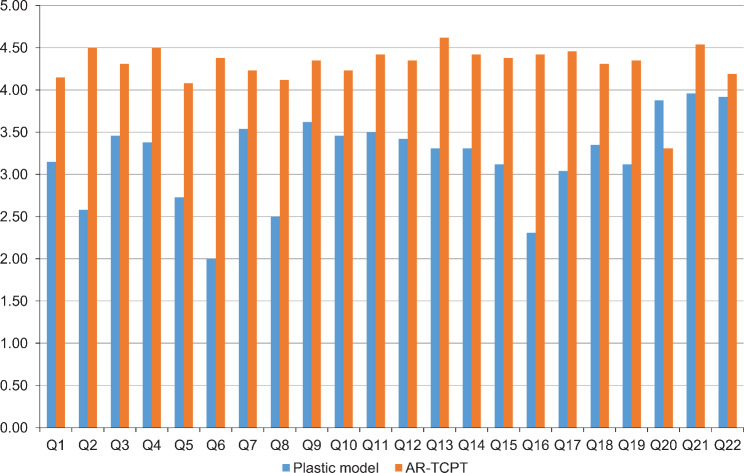
Comparison of questionnaire scores. Bar chart comparing the mean scores of the control group using the plastic model and the experimental group using the augmented reality application. AR-TCPT, augmented reality-based tooth carving practice tool

Table 2 presents the descriptive statistics and statistical comparisons of responses to the 22 survey items between the groups. The responses to each of the 22 items in the questionairre differed significantly between groups (p < 0.01). Compared with the control group, the experimental group had a higher mean score for 21 questionnaire items. Only in question 20 (Q20) of the questionnaire did the control group score higher than the experimental group. The difference in mean scores between groups is visually depicted in the bar chart in Fig. 7. Table 2; Fig. 7 also show the evaluation results of user experience for each item. In the control group, the highest-rated item was Q21, whereas the lowest-rated item was Q6. In the experimental group, the highest-rated item was Q13, whereas the lowest-rated item was Q20. As shown in Fig. 7, the largest difference in mean values between the control and experimental groups was observed in Q6, whereas the smallest difference was in Q22.

**Table 2:** Comparison of all questionnaire item responses between the control and experimental groups

Q	Group	N	Descriptive statistics			Test statistics from Mann–Whitney U tests				
			M ± SD	Median		Mean rank	Sum of ranks	*U*	*z*	*p*-value
Q1	CG	26	3.15 ± 1.01	3.0		19.37	503.50	152.5	-3.56	0.000**
	EG	26	4.15 ± 0.78	4.0		33.63	874.50			
Q2	CG	26	2.58 ± 0.81	3.0		14.88	387.00	36.0	-5.71	0.000**
	EG	26	4.50 ± 0.71	5.0		38.12	991.00			
Q3	CG	26	3.46 ± 0.76	4.0		19.00	494.00	143.0	-3.96	0.000**
	EG	26	4.31 ± 0.55	4.0		34.00	884.00			
Q4	CG	26	3.38 ± 0.70	3.5		17.13	445.50	94.5	-4.73	0.000**
	EG	26	4.50 ± 0.65	5.0		35.87	932.50			
Q5	CG	26	2.73 ± 0.87	3.0		17.08	444.00	93.0	-4.67	0.000**
	EG	26	4.08 ± 0.74	4.0		35.92	934.00			
Q6	CG	26	2.0 ± 0.85	2.0		13.85	360.00	9.0	-6.15	0.000**
	EG	26	4.38 ± 0.64	4.0		39.15	1018.00			
Q7	CG	26	3.54 ± 0.81	4.0		20.85	542.00	191.0	-2.95	0.003*
	EG	26	4.23 ± 0.71	4.0		32.15	836.00			
Q8	CG	26	2.50 ± 0.95	2.5		16.50	429.00	78.0	-4.91	0.000**
	EG	26	4.12 ± 0.86	4.0		36.50	949.00			
Q9	CG	26	3.62 ± 0.90	4.0		20.63	536.50	185.5	-2.97	0.003*
	EG	26	4.35 ± 0.69	4.0		32.37	841.50			
Q10	CG	26	3.46 ± 0.65	4.0		19.65	511.00	160.0	-3.54	0.000**
	EG	26	4.23 ± 0.71	4.0		33.35	867.00			
Q11	CG	26	3.50 ± 0.71	4.0		18.25	474.50	123.5	-4.36	0.000**
	EG	26	4.42 ± 0.58	4.0		34.75	903.50			
Q12	CG	26	3.42 ± 0.70	4.0		18.73	487.00	136.0	-3.91	0.000**
	EG	26	4.35 ± 0.85	5.0		34.27	891.00			
Q13	CG	26	3.31 ± 0.79	3.5		16.31	424.00	73.0	-5.11	0.000**
	EG	26	4.62 ± 0.64	5.0		36.69	954.00			
Q14	CG	26	3.31 ± 0.79	3.0		17.38	452.00	101.0	-4.62	0.000**
	EG	26	4.42 ± 0.64	4.5		35.62	926.00			
Q15	CG	26	3.12 ± 0.91	3.0		17.04	443.00	92.0	-4.82	0.000**
	EG	26	4.38 ± 0.57	4.0		35.96	935.00			
Q16	CG	26	2.31 ± 0.79	2.5		14.65	381.00	30.0	-5.81	0.000**
	EG	26	4.42 ± 0.81	5.0		38.35	997.00			
Q17	CG	26	3.04 ± 0.82	3.0		16.62	432.00	81.0	-4.88	0.000**
	EG	26	4.46 ± 0.76	5.0		36.38	946.00			
Q18	CG	26	3.35 ± 0.69	3.0		18.75	487.50	136.5	-3.87	0.000**
	EG	26	4.31 ± 0.88	5.0		34.25	890.50			
Q19	CG	26	3.12 ± 0.95	3.0		17.44	453.50	102.5	-4.59	0.000**
	EG	26	4.35 ± 0.63	4.0		35.56	924.50			
Q20	CG	26	3.88 ± 0.65	4.0		31.75	825.50	201.5	-2.75	0.006*
	EG	26	3.31 ± 0.68	3.0		21.25	552.50			
Q21	CG	26	3.96 ± 0.60	4.0		20.37	529.50	178.5	-3.24	0.001*
	EG	26	4.54 ± 0.58	5.0		32.63	848.50			
Q22	CG	26	3.92 ± 0.27	4.0		23.19	603.00	252.0	-2.66	0.008*
	EG	26	4.19 ± 0.40	4.0		29.81	775.00			

*p < 0.01, **p < 0.001

CG, control group; EG, experimental group; M, mean; N, number of participants; Q, questionnaire items; SD, standard deviation

## Discussion

AR technology has become increasingly popular in various areas of dentistry, including clinical aesthetics, maxillofacial surgery, restorative techniques, dental morphology and implantology, and simulation [28–31]. For instance, Microsoft HoloLens provides advanced AR tools that augment dental education and surgical planning [32]. Virtual reality technology also provides a simulated environment for dental morphological education [33]. Although advanced hardware-dependent head-mounted displays for these technologies are not yet widespread in dental education, AR mobile applications can enhance clinical application skills and help users quickly understand anatomical structures [34, 35]. AR technology can also increase students’ motivation and interest in learning dental morphology, providing a more interactive and engaging learning experience [36]. AR learning tools help students visualize complex dental procedures and anatomy in 3D [37], which is critical for understanding tooth morphology.

The effect of 3D-printed plastic tooth models on tooth morphology education has been superior to that of textbooks with 2D pictures and explanations [38]. However, digitization of education and technological advancements have made it imperative to introduce various devices and technologies in health and medical education, including dental education [35]. Instructors face challenges teaching complex concepts in a rapidly evolving and dynamic field [39], and using a variety of practice media, in addition to traditional plastic models of teeth, is necessary to aid students in tooth carving practice. Therefore, the present study introduced a practice tool, the AR-TCPT, that ultizes AR technology to aid dental morphology practice.

Investigating the user experience of AR applications is crucial for understanding the factors that impact media utilization [40]. Positive user experiences of AR can guide the direction and improvement of its development, including its purpose, ease of use, smooth operation, information display, and interaction [41]. As shown in Table 2, the experimental group using the AR-TCPT received higher user experience scores compared with those of the control group using plastic models, except for Q20. Compared with the plastic model, the AR-TCPT received high ratings for its user experience in tooth carving practice. The ratings included understanding, visualization, observation, repetition, tool usefulness, and perspective variety. The benefits of using the AR-TCPT included quick comprehension, efficient navigation, time-saving, preclinical carving skill development, comprehensive coverage, improved learning, reduced reliance on textbooks, and the interactive, enjoyable, and informative nature of the experience. The AR-TCPT also facilitated easy interaction with other practice tools and provided clear views from multiple angles.

As shown in Fig. 7, the AR-TCPT has a supplementary point presented in Q20: a comprehensive graphical user interface displaying all tooth carving steps is required to assist learners in tooth carving. The display of the entire tooth carving process is fundamental in developing tooth carving skills before treating patients. The experimental group gave their highest rating in Q13, a fundamental question related to aiding the development of tooth carving skills prior to treating patients and enhancing the user’s tooth carving skill, highlighting the potential of the tool in tooth carving practice. The user expects to apply the learned skills in a clinical setting. However, follow-up studies are necessary to evaluate the development and effectiveness of actual tooth carving skills. Q6 asked whether plastic models and the AR-TCTP were usable when necessary, with respones to this question showing the biggest difference between the two groups. As a mobile application, the AR-TCPT was confirmed to have higher usability compared with the plastic model. However, it remains challenging to prove the educational efficacy of AR applications solely through user experience. Further studies are necessary to evaluate the impact of AR-TCTP on finished tooth pieces. Nevertheless, in the present study, the high user experience score for the AR-TCPT suggested its potential as a practice tool.

This comparative study suggests that the AR-TCPT has the potential to be a valuable alternative or complementary tool to the conventional plastic model for dental practice, as it received a superior evaluation score for user experience. However, to establish its superiority, further quantitative evaluations by instructors are required for intermediate and final carved teeth. Moreover, the impact of individual differences in spatial perception ability on the carving process and the final teeth must be analyzed. Dental ability varies among individuals, which may affect the carving process and final teeth. Therefore, additional research is needed to demonstrate the effectiveness of the AR-TCPT as a practice tool for tooth carving and understand the regulatory and mediating roles of AR applications in the carving process. Future studies should focus on evaluating the developmental and evaluative aspects of practice tools using advanced HoloLens AR technology for dental morphology.

## Conclusions

In conclusion, this study demonstrated the potential of the AR-TCPT as a practice tool for tooth carving, as it provided an innovative and interactive learning experience for students. Compared with the conventional plastic model group, the AR-TCPT group showed significantly higher scores in user experience, including benefits such as quick comprehension, improved learning, and reduced reliance on textbooks. With its familiar technology and ease of use, the AR-TCPT offers a promising alternative to conventional plastic tools and may assist beginners struggling with 3D carving. However, further research is required to evaluate its educational efficacy, including its effects on individual carving abilities and quantitative evaluations of carved teeth.

## Data Availability

The datasets utilized in this study can be obtained by contacting the corresponding author through a reasonable request.

## List of abbreviations

2D: Two-dimensional
3D: Three-dimensional
AR: Augmented reality
AR-TCPT: Augmented reality-based tooth carving practice tool

## References

[1] Bogacki RE, Best A, Abbey LM (2004). Equivalence study of a dental anatomy computer-assisted learning program. J Dent Educ.

[2] Kellesarian SV (2018). Flipping the dental anatomy classroom. Dent J (Basel).

[3] Abu Eid R, Ewan K, Foley J, Oweis Y, Jayasinghe J (2013). Self–directed study and carving tooth models for learning tooth morphology: perceptions of students at the University of Aberdeen, Scotland. J Dent Educ.

[4] Lone M, McKenna JP, Cryan JF, Downer EJ, Toulouse A (2018). A survey of tooth morphology teaching methods employed in the United Kingdom and Ireland. Eur J Dent Educ.

[5] Obrez A, Briggs C, Buckman J, Goldstein L, Lamb C, Knight WG (2011). Teaching clinically relevant dental anatomy in the dental curriculum: description and assessment of an innovative module. J Dent Educ.

[6] Costa AK, Xavier TA, Paes-Junior TJ, Andreatta-Filho OD, Borges AL (2014). Influence of occlusal contact area on cusp defection and stress distribution. J Contemp Dent Pract.

[7] Shugars DA, Bader JD, Phillips SW, White BA, Brantley CF (2000). The consequences of not replacing a missing posterior tooth. J Am Dent Assoc.

[8] Wang H, Xu H, Zhang J, Yu S, Wang M, Qiu J (2020). The effect of 3D-printed plastic teeth on scores in a tooth morphology course in a chinese university. BMC Med Educ.

[9] Risnes S, Khan Q, Hadler-Olsen E, Sehic A (2019). Tooth identification puzzle: a method of teaching and learning tooth morphology. Eur J Dent Educ.

[10] Kirkup ML, Adams BN, Reifeis PE, Heselbarth JL, Willis LH (2019). Is a picture worth a thousand words? Effectiveness of iPad technology in preclinical dental laboratory courses. J Dent Educ.

[11] Goodacre CJ, Younan R, Kearbey V, Fitzpatrick M (2021). An educational experiment resulting from COVID-19: the use of at‐home waxing and webinars for teaching a 3‐week intensive course in tooth morphology to first year dental students. J Prosthodont.

[12] Roy E, Bakr MM, George R (2017). The need for virtual reality simulators in dental education: a review. Saudi Dent J.

[13] Azuma RT (1997). A survey of augmented reality. Presence Teleoperators Virtual Environ.

[14] Garzón J (2021). An overview of twenty-five years of augmented reality in education. Multimodal Technol Interact.

[15] Tan SY, Arshad H, Abdullah A (2018). An efficient and robust mobile augmented reality application. Int J Adv Sci Eng Inf Technol.

[16] Wang M, Callaghan V, Bernhardt J, White K, Peña-Rios A (2018). Augmented reality in education and training: pedagogical approaches and illustrative case studies. J Ambient Intell Human Comput.

[17] Pellas N, Fotaris P, Kazanidis I, Wells D (2019). Augmenting the learning experience in primary and secondary school education: a systematic review of recent trends in augmented reality game-based learning. Virtual Real.

[18] Mazzuco A, Krassmann AL, Reategui E, Gomes RS (2022). A systematic review of augmented reality in chemistry education. Rev Educ.

[19] Akçayır M, Akçayır G (2017). Advantages and challenges associated with augmented reality for education: a systematic review of the literature. Educ Res Rev.

[20] Dunleavy M, Dede C, Mitchell R (2009). Affordances and limitations of immersive participatory augmented reality simulations for teaching and learning. J Sci Educ Technol.

[21] Cheng KH, Tsai CC (2013). Affordances of augmented reality in science learning: suggestions for future research. J Sci Educ Technol.

[22] Kilistoff AJ, Mackenzie L, D’Eon M, Trinder K (2013). Efficacy of a step-by-step carving technique for dental students. J Dent Educ.

[23] Wang S, Zhao W, Ye H, Liu Y, Zhou Y. Preliminary application and evaluation of digital step-by-step tooth-preparation templates. J Prosthet Dent. 2021. 10.1016/j.prosdent.2021.09.009 34702585

[24] Yuan JX, Yang KY, Ma J, Wang ZZ, Guo QY, Liu F (2020). Step-by-step teaching method: improving learning outcomes of undergraduate dental students in layering techniques for direct composite resin restorations. BMC Med Educ.

[25] Liu X, Liu M, Yang Y, Fan C, Tan J (2019). Step-by-step teaching method improves the learner achievement in dental skill training. Eur J Dent Educ.

[26] Saunders M, Lewis P, Thornhill A. Research methods for business students. Pearson Education; 2009.

[27] Alfalah SFM, Falah JFM, Alfalah T, Elfalah M, Muhaidat N, Falah O (2019). A comparative study between a virtual reality heart anatomy system and traditional medical teaching modalities. Virtual Real.

[28] Fahim S, Maqsood A, Das G, Ahmed N, Saquib S, Lal A (2022). Augmented reality and virtual reality in dentistry: highlights from the current research. Appl Sci.

[29] Haji Z, Arif A, Jamal S, Ghafoor R (2021). Augmented reality in clinical dental training and education. JPMA.

[30] Mladenovic R. The usage of augmented reality in dental education. Augmented Real Educ. 2020;139–57.

[31] Joda T, Gallucci GO, Wismeijer D, Zitzmann NU (2019). Augmented and virtual reality in dental medicine: a systematic review. Comput Biol Med.

[32] Blanchard J, Koshal S, Morley S, McGurk M (2022). The use of mixed reality in dentistry. Br Dent J.

[33] Liebermann A, Seefelder JK, Huth KC, Erdelt K (2023). Mobile virtual tooth morphology teaching environment for preclinical dental students. J Dent Educ.

[34] Juan M, Alexandrescu L, Folguera F, García García I (2016). A mobile augmented reality system for the learning of dental morphology. DER.

[35] Gredes T, Pricop-Jeckstadt M, Mereti E, Botzenhart U (2022). Survey of student attitudes toward digital technology in practical technical dental education using the AR‐Demonstrator‐App. J Dent Educ.

[36] Monterubbianesi R, Tosco V, Vitiello F, Orilis G, Fraccastoro F, Putignano A, Orsini G (2022). Augmented, virtual and mixed reality in dentistry: a narrative review on the existing platforms and future challenges. Appl Sci.

[37] Dzyuba N, Jandu J, Yates J, Kushnerev E. Virtual and augmented reality in dental education: the good, the bad and the better. Eur J Dent Educ. 2022. 10.1111/eje.12871 PMC12287989 36336847

[38] Wang H, Xu H, Zhang J, Yu S, Wang M, Qiu J, Zhang M (2020). The effect of 3D-printed plastic teeth on scores in a tooth morphology course in a chinese university. BMC Med Educ.

[39] Moro C, Smith J, Stromberga Z (2019). Multimodal learning in health sciences and medicine: merging technologies to enhance student learning and communication. Biomed Vis.

[40] Satkowski M, Büschel W, Dachselt R. Experiences with user studies in augmented reality. arXiv preprint arXiv:2104.03795. 2021.

[41] Davidavičienė V, Raudeliūnienė J, Viršilaitė R (2020). Evaluation of user experience in augmented reality mobile applications. J Bus Econ Manag.

